# Unveiling the complex phonon nature and phonon cascades in 1L to 5L WSe_2_ using multiwavelength excitation Raman scattering†

**DOI:** 10.1039/d4na00399c

**Published:** 2024-07-30

**Authors:** Claire Blaga, Ángel Labordet Álvarez, Akshay Balgarkashi, Mitali Banerjee, Anna Fontcuberta i Morral, Mirjana Dimitrievska

**Affiliations:** a Laboratory of Semiconductor Materials, Institute of Materials, School of Engineering, École Polytechnique Fédérale de Lausanne 1015 Lausanne Switzerland; b Nanomaterials Spectroscopy and Imaging Group, Transport at Nanoscale Interfaces Laboratory, Swiss Federal Laboratories for Material Science and Technology (EMPA) Ueberlandstrasse 129 8600 Duebendorf Switzerland mirjana.dimitrievska@empa.ch; c Laboratory of Quantum Physics, Topology and Correlations, Institute of Physics, School of Basic Sciences, Ecole Polytechnique Fédérale de Lausanne 1015 Lausanne Switzerland; d Institute of Physics, School of Basic Sciences, Ecole Polytechnique Fédérale de Lausanne 1015 Lausanne Switzerland

## Abstract

Tungsten diselenide (WSe_2_) is a 2D semiconducting material, promising for novel optoelectronic and phononic applications. WSe_2_ has complex lattice dynamics and phonon structure. Numerous discrepancies in the literature exist regarding the interpretation and identification of phonon modes. This work presents a complete investigation of the vibrational properties of 1L to 5L flakes and bulk WSe_2_ using multi-wavelength Raman spectroscopy. We especially highlight measurements using 785 nm excitation, which have not been performed before. These allow us to solve inconsistences in the literature in terms of defect-activated non-*Γ* point single phonon modes and Breit–Wigner–Fano type resonance. We identify 35 Raman peaks per flake thickness, which we attribute to either one-phonon or multi-phonon modes, including two-phonon scattering due to a van Hove singularity (vHs). The measurements are in excellent agreement with the theoretical predictions. Using photoluminescence measurements, we identify photon-exciton coupling leading to resonant Raman scattering, suggesting wavelength laser excitations best suited for further investigations of specific WSe_2_ flake thicknesses. Finally, we report the observation of phonon-cascades for all WSe_2_ flake thicknesses, indicating strong phonon–electron interactions during early carrier relaxation processes in WSe_2_. This research provides a solid foundation and reference for future investigations of the vibrational properties of WSe_2_, paving the way for further development of this material towards applications.

## Abstract 2

We unveil the complex phonon dynamics of WSe_2_ by using multiwavelength excitation Raman spectroscopy and report phonon-cascades for all WSe_2_ flake thicknesses, indicating strong phonon–electron interactions during early carrier relaxation processes.

## Graphical abstract

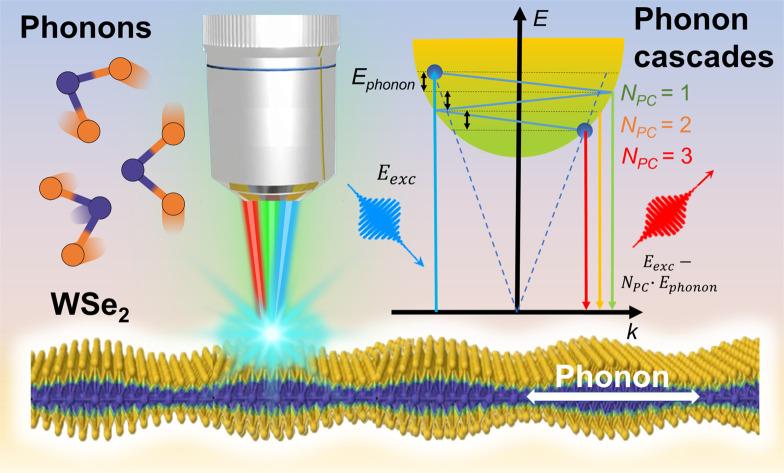

## Introduction

Research on two-dimensional (2D) materials, ignited by the discovery of graphene, has led to the revelation of a multitude of layered materials with promising properties. The family of transition metal dichalcogenides (TMDs) is of particular significance. TMDs are composed of three atom-thick monolayers, made up of a transition metal atom layer sandwiched between two chalcogen atom layers. Unlike graphene, some TMDs, such as MoS_2_ and WSe_2_, are semiconductors with bandgaps in the visible range (1–2.5 eV).^1–3^ They exhibit thickness-dependent properties resulting from symmetry breaking and quantum confinement. WSe_2_ and MoS_2_ show an indirect to direct bandgap transition when going from multi- to monolayer thickness, exhibiting bright photoluminescence from strongly bound excitons in the latter case.^1^ TMDs host valley-selective excitons, opening a door towards valley-based optoelectronic engineering.^4^ WSe_2_, in particular, has been shown to host deterministically engineered single photon emission.^5^ Despite being ideal candidates for many ultra-thin optoelectronic applications, there are still open questions concerning their phononic properties.

Understanding the vibrational (phononic) properties of thin layer TMDs is crucial for applications requiring the engineering of thermal conductivity.^6–8^ It is also essential for leveraging the interlayer charge transfer dynamics in van der Waals heterostructures,^9,10^ particularly for optoelectronic and photovoltaic applications, and the investigation of Moiré phonons in twisted layers of TMDs for novel phononic devices.^11^

Raman spectroscopy is a routinely used technique for the characterization of 2D materials, as it provides information on the structure,^12,13^ defect type and density,^14,15^ and strain of the lattice.^16–18^ It also gives access to a variety of electronic parameters such as the charge carrier density,^19,20^ maximum charge carrier mobility,^17,21^ phonon–electron coupling and electron–electron scattering.^22,23^ Considering the plethora of information that Raman spectroscopy can provide, a precise comprehension of the Raman spectra and the lattice dynamics is crucial for their further development and utilization in future applications.

Vibrational properties of few-layered WSe_2_ have been explored in several studies before, where most of the effort was made on the proper interpretation of Raman peaks of WSe_2_.^24–32^ Many of these studies show significant discrepancies in the analysis of the Raman spectra and assignment of phonon modes. For example, the interpretation of Raman peaks and assigned phonon modes vary across the literature, from 17 to 42 phonon modes observed.^24–32^ Interestingly, several studies report activation of non-*Γ* point single phonon modes in the Raman spectra of WSe_2_ flakes.^30,33,34^ These modes are usually not expected in the Raman spectrum of high crystal quality materials, as they require the presence of defects in order to be activated.^35–37^ Some studies report the observation of the Breit–Wigner–Fano (BWF) type resonance of the low intensity Raman peak at 302 cm^−1^ assigned to WSe_2_.^30^ This is very unusual, as theoretically calculated phonon frequencies do not predict the presence of any single or multi-phonon mode at this position. Finally, one finds different interpretations of the Raman peak at 260 cm^−1^, from the identification as a broad 2LA(M) mode, to the deconvolution into a dozen peaks assigned to one-phonon and multi-phonon modes.^33,34,38^ Such inconsistencies in reported results require clarification by a systematic review of published data and an in-depth investigation of vibrational properties of multilayer WSe_2_ systems.

In this work, we investigate vibrational properties of multilayer (1L to 5L) and bulk WSe_2_ using multiwavelength excitation Raman spectroscopy. Using a rigorous deconvolution process of the Raman spectra measured with 488, 532 and 785 nm laser excitations, we provide a complete analysis of all Raman active modes of WSe_2_ systems. To the best of our knowledge, this is the first comprehensive analysis of the Raman spectra of WSe_2_ measured at 785 nm, along with a concise comparison of theoretical and experimental results in terms of phonon symmetries and frequencies. We also shed new light on the inconsistencies reported in the literature regarding the activation of non-*Γ* point single phonon modes and BWF type resonance. Finally, we highlight the observation of the phonon cascades in 1L to 5L and bulk WSe_2_ as well as suggest the optimal laser excitations under which different numbers of layers could be further explored. These results can be used as a reference for future studies of vibrational properties of WSe_2_, paving the way for successful integration of WSe_2_ in phononic and optoelectronic devices.

## Materials and methods

### Material preparation

The multi-layered WSe_2_ flake was obtained by mechanical exfoliation by thinning down a crystal of bulk WSe_2_ (HQ graphene) with Scotch tape. The thinned crystals were transferred onto a silicon chip covered with 285 nm of SiO_2_ (NOVA Electronic Materials) using Scotch tape.

### Characterization

Raman and photoluminescence (PL) measurements were performed on a WITec Alpha 300 R confocal Raman microscope in backscattering geometry. Multiwavelength excitation Raman measurements were performed using 488, 532, and 785 nm lasers. The beam was focused on the sample with a microscope objective, resulting in a diameter spot of 800 nm for the 488 nm laser, 1 μm for the 532 nm laser, and 1.2 μm for the 785 nm laser, and reaching a radiant output power of the order of 500 μW, equivalent to 150 μW at the sample. Laser power conditions were selected based on a power study that measured the Raman spectrum at the same point on the material with increasing laser power densities, starting from the lowest power available. For each laser power, the spectrum was monitored for changes in peak positions, peak widths, or the appearance of new peaks. The highest power for which no changes in these parameters were observed was taken as the optimal laser power for measurements. The backscattered light was analyzed with two spectrometers: a 300 mm lens-based spectrometer with a grating of 1800 g mm^−1^ equipped with a thermoelectrically cooled CCD for 488 and 532 nm excitation and a 400 mm lens-based spectrometer with a grating of 1200 g mm^−1^ equipped with a cooled deep-depletion CCD for 785 nm excitation. All Raman peak positions were calibrated with respect to the Si Raman peak at 520 cm^−1^.

Atomic force microscopy (AFM) measurements were performed on a Bruker FastScan AFM, using a ScanAsyst_Fluid+ tip. The AFM mapping is performed in tapping-mode to ensure that the flake does not incur any damage from contact with the tip.

The optical microscopy images were taken with a BX53M Olympus microscope, using a 6.4 megapixel DP23 Olympus digital color camera and an LMPLFLN100x objective.

## Results and discussion

### Morphological assessment of the WSe_2_ flake

**Fig. 1:**
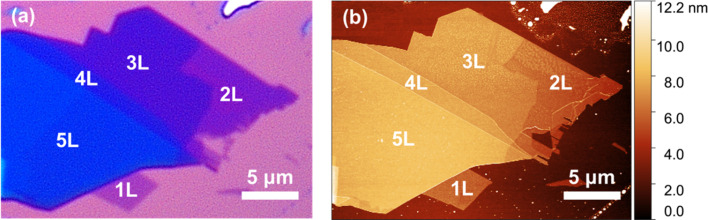
Morphological characterization of a WSe_2_ flake on a SiO_2_/Si substrate. (a) Optical microscopy image showing different contrasts within the WSe_2_ flake attributed to different numbers of layers: monolayer (1L), bilayer (2L), three-layer (3L), four-layer (4L) and five-layer (5L). (b) AFM image of the WSe_2_ flake measured in tapping-mode showing the height profile and confirming the assignment of the different numbers of layers.

The morphological characterization of the WSe_2_ flake, as well as the determination of the number of layers, was performed using optical microscopy, AFM and Raman imaging. Fig. 1a shows the optical microscopy image of the flake prior to any further characterization. The optical contrast can be used to empirically determine the thickness of the different parts of the flake. The thickness of the flake is directly correlated with its transparency, with thinner areas being more transparent. The exact number of layers is determined from AFM measurements, which reliably allows to discriminate between different thicknesses as shown in Fig. 1b. This allowed determination of the regions of monolayer (1L), bilayer (2L), three-layer (3L), four-layer (4L) and five-layer (5L) as labeled in Fig. 1. The height profiles of the different layers confirming this assignment are shown in Fig. S1 in the ESI.†

**Fig. 2:**
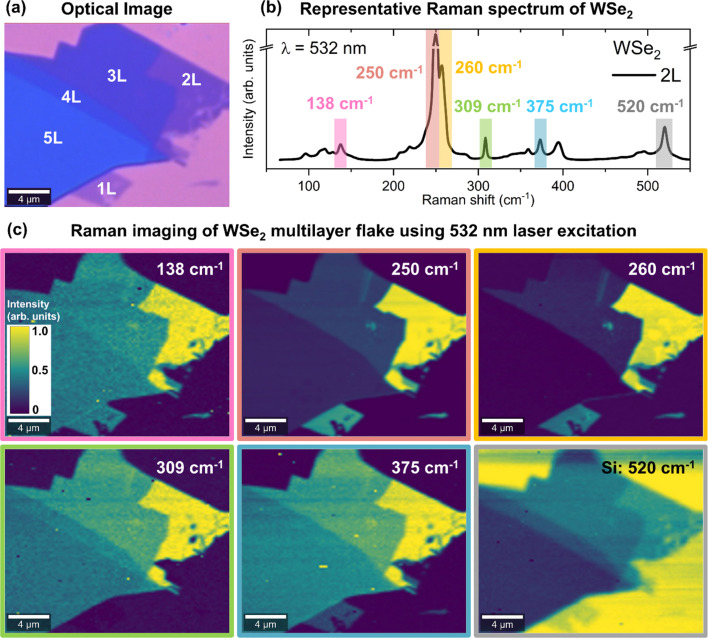
Raman imaging of the multilayer WSe_2_ flake. (a) Optical image of the flake using a Raman microscope. (b) Representative spectrum of the WSe_2_ flake recorded on the bilayer area using an excitation wavelength of 532 nm. The main peaks are highlighted on the spectrum and their respective intensities over the flake are shown in the Raman images in (c).

In order to confirm the homogeneity and high crystal quality of the WSe_2_ flake, we have performed high-resolution Raman imaging using 532 nm laser excitation with a laser spot diameter of 1 μm, as shown in Fig. 2. It should be noted that before any Raman measurements, a laser power study was performed on the small area of the monolayer, which was used to determine the maximal laser power. A consequence of the power study is structural damage to the small area of the monolayer, which is noticeable by the dark spot in Fig. 2a. This area was excluded from any further analysis that was performed in this study.

Fig. 2b shows a representative Raman spectrum of bilayer (2L) WSe_2_, highlighting some of the most intense Raman peaks at 138, 250, 260, 309 and 375 cm^−1^ of WSe_2_, as well as the 520 cm^−1^ peak corresponding to the Si substrate. Variations in the Raman intensity of these peaks over the WSe_2_ flake are shown in the Raman mapping images in Fig. 2c. It can be observed that there is no notable change in the intensity of Raman peaks within each area of a single thickness, for all presented Raman maps. This signals structural uniformity of layers. Additionally, we can observe that the Raman intensity variation between different layers changes depending on which Raman peak is used for imaging. For example, Raman maps corresponding to the intensity of 250 and 260 cm^−1^ peaks show clear differences in the 1L and 2L areas. The difference between 3L, 4L and 5L is harder to distinguish. On the other hand, the Raman intensity maps corresponding to 309 and 375 cm^−1^ peaks show the difference among 2L, 3L, 4L and 5L better, while making the identification of 1L areas difficult. Indeed, in the case of 1L, the 375 cm^−1^ peak has very low intensity and the 309 cm^−1^ peak is not active. In contrast, Raman imagining with low frequency peaks, such as 138 cm^−1^, does not show any major differences in the areas corresponding to different numbers of layers. Interestingly, the best imaging of the differences in the number of layers is achieved with Raman mapping of the Si peak at 520 cm^−1^, enabling clear differentiation between 1L and 5L areas. We attribute this to the absorbance of the Si Raman emission by the layers, similarly to the work of Negri *et al.*^39^ This illustrates the potential of using Raman imaging as a complementary method for the identification of the number of layers, when AFM measurements are not possible. However, care should be taken when selecting the Raman peaks used for imaging, depending on the number of layers that need to be identified.

### Phonon modes and phonon dispersion in multilayer WSe_2_

Bulk 2H-WSe_2_ belongs to the space group *D*^4^_6h_ (*P*6_3_/*mmc*) with the unit cell containing two W and four Se atoms. Group theory analysis predicts the following set of irreducible representations at the *Γ* point of the Brillouin zone:^40–42^*Γ*_total_ = A_1g_ + 2A_2u_ + 2B_2g_ + B_1u_ + E_1g_ + 2E_1u_ + 2E_2g_ + E_2u_.

Raman and infra-red active modes are:*Γ*_Raman_ = A_1g_ + E_1g_ + 2E_2g_*Γ*_IR_ = 2A_2u_ + 2E_1u_while B_2g_, B_1u_ and E_2u_ modes are inactive phonon modes. Also note that A and B refer to the non-degenerate symmetric and asymmetric modes with respect to the principal symmetry axis, while E refers to double degenerate modes. The distinctions g and u correspond to symmetric or asymmetric vibrations with respect to the center of inversion.

In contrast to the bulk, multilayer WSe_2_ systems are considered quasi 2D, due to the lack of translation symmetry along the *c*-axis direction. Their space group symmetry is determined by the parity of the number of layers. An odd number of layers is classified within the *D*_3h_ point group symmetry (without inversion symmetry), while an even number of layers belongs to the *D*_3d_ point group symmetry (with a center of inversion). The labeling of phonon modes is different depending on the number of layers, and the irreducible representations at the *Γ* point of the Brillouin zone are:^40–42^
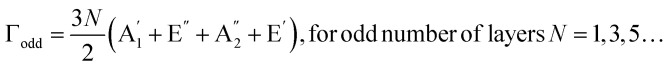

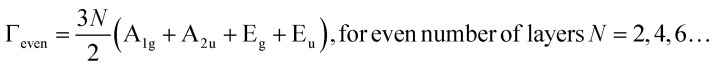


Therefore, strictly speaking, the notations of the modes for the multilayer WSe_2_ systems differ, with A_1g_ and E_2g_ modes for bulk, translating to 
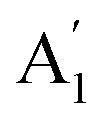
 and E′ modes for an odd number of layers and A_1g_ and E_g_ modes for an even number of layers.

The one-phonon modes described above, arising from the *Γ* point, are a result of the first-order Raman scattering process. Higher-order Raman scattering processes are also possible. These involve the combination of multiple phonon modes from *Γ* and non-*Γ* points of the Brillouin zone for which the conservation of the momentum rule is preserved. This means combining two (or more) phonons from the *Γ* point which have zero momentum, or combining phonons from *M* and *K* points (or anywhere else in the Brillouin zone) whose total momentum is zero (for example pairs of phonons with equal and opposite momentum). Higher-order Raman scattering is less probable than the first-order, and therefore the combination modes in the Raman spectrum have lower intensities and higher widths of peaks when compared to the first-order Raman modes. These intensities can be significantly enhanced under Raman resonant conditions, when the excitation wavelength is close to the energy of some interband transition in the material. Therefore, for a complete interpretation of the Raman spectrum, it is important to consider the full phonon and energy band dispersion of the material, over the whole Brillouin zone.

The band structure and phonon dispersions of bulk and multilayer WSe_2_ systems have been extensively studied and reported in the literature.^25,27–29,33,34,43–45^ For convenience, Fig. S2 in the ESI† presents the density functional theory (DFT) calculated phonon dispersions of monolayer (1L) WSe_2_ adapted from ref. 33. Monolayer WSe_2_ has three atoms per unit cell, resulting in a total of nine phonons. The three acoustic phonons correspond to the out-of-plane (ZA) vibrations, in-plane transverse (TA) vibrations, and in-plane longitudinal (LA) vibrations. These phonons are located at 0 cm^−1^ at the *Γ* point and correspond to the lowest frequency phonon branches across the Brillouin zone in Fig. S2.† The E′′ and E′ phonon modes are located at 176 and 250 cm^−1^ at the *Γ* point, and as doubly degenerated modes, they are split into two optical branches each, over the Brillouin zone. The lower frequency phonon branches correspond to transverse (TO_1_ and TO_2_) vibrations, while the higher frequency phonon branches correspond to longitudinal (LO_1_ and LO_2_) vibrations of E′′ and E′ modes, respectively. The 
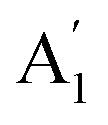
 and 
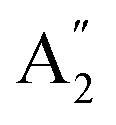
 modes are located at 250 and 310 cm^−1^ at the *Γ* point and correspond to two out of plane (ZO_1_ and ZO_2_) vibrations, respectively. They are represented by the highest frequency phonon branches in Fig. S2.†

In comparison, the phonon dispersions of the multilayer WSe_2_ systems (2L, 3L and higher) slightly differ from those of the monolayer WSe_2_. The main difference is in the number of expected phonons, *i.e.* phonon branches, with the total number of phonons being calculated as 3*N*, where *N* is the number of atoms per unit cell. Therefore, bilayer (2L) WSe_2_, having 6 atoms per unit cell, will have a total of 18 phonon branches, while trilayer (3L) WSe_2_, with 9 atoms per unit cell, will have a total of 27 phonon branches, *etc.* The new phonon branches in the multilayer systems arise from the splitting of the monolayer phonon branches. Therefore, the frequencies of these new phonons are very close to the one of the original monolayer phonon branch. This is the main reason why the overall phonon dispersion of WSe_2_ looks very similar regardless of the number of layers.^33^ Another difference is the appearance of the low frequency shear and breathing modes, which arise due to the interlayer van der Waals (vdW) coupling between the layers. Their frequencies depend on the number of layers present.^43^

It should be noted that the DFT predicted frequencies of the phonon modes, especially at *Γ*, *M* and *K* points of the Brillouin zone, are very important and will be used for the identification of the combination phonon modes in the Raman spectra of WSe_2_ multilayer systems.

### Raman spectra and phonon mode identification in multilayer and bulk WSe_2_

**Fig. 3:**
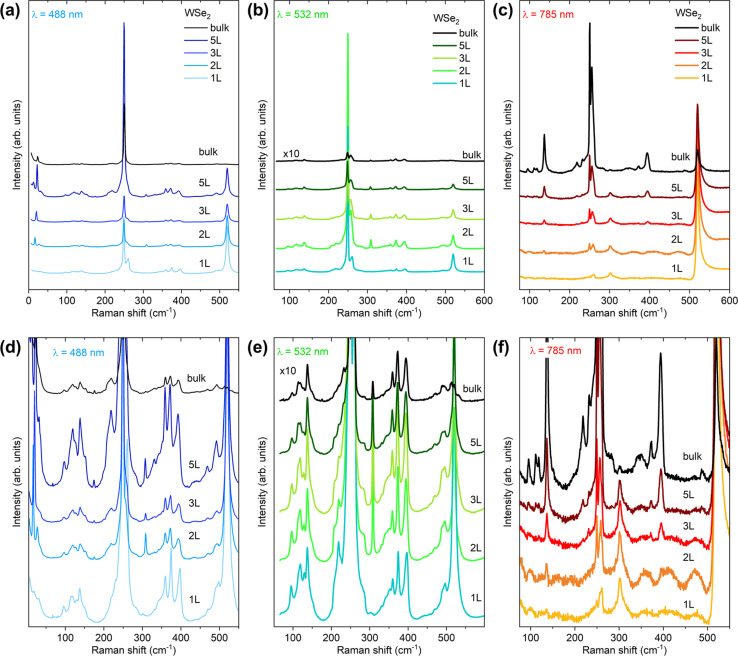
Raman spectra of 1–5L and bulk WSe_2_ measured with (a and d) 488 nm, (b and e) 532 nm and (c and f) 785 nm wavelength excitations. The top panel (a–c) presents the Raman spectra at the absolute intensity scale, while the bottom panel (d–f) presents a magnified intensity scale, allowing observation of low intensity Raman active peaks. The measurements were performed at room temperature and under the same acquisition conditions per excitation wavelength, allowing direct comparison of the Raman intensity among the different numbers of WSe_2_ layers.

Fig. 3 shows the Raman spectra of 1L–5L and bulk WSe_2_ measured at 488, 532 and 785 nm excitation wavelengths. The measurements were performed at room temperature under the same acquisition conditions for all layers, including density of laser power and acquisition times. This allows for comparison of the absolute Raman intensities among the different layers and enables identification of conditions leading to a significant increase in intensity, for example close to Raman resonant conditions. Fig. 3a–c show the comparison of Raman spectra at the absolute intensity scale, while Fig. 3d–e present a magnified intensity scale, allowing better observation of all Raman active peaks. Large differences in the shape of the Raman spectra with changes in the laser wavelength are observed. The highest intensity of Raman peaks is seen for 5L, 2L and bulk when measured with 488, 532 and 785 nm wavelength excitations, respectively. Additionally, we observe that spectral fingerprints, in the frequency regions from 50 to 200 cm^−1^, look similar for 488 and 532 nm laser excitations, while a significant difference is seen in the case of 785 nm excitation. A similar conclusion can be made for the frequency region from 300 to 500 cm^−1^. On the other hand, the spectral features do not change significantly with the change in the number of layers. The main difference in this case is observed in the change in the intensity of the peaks.

**Fig. 4:**
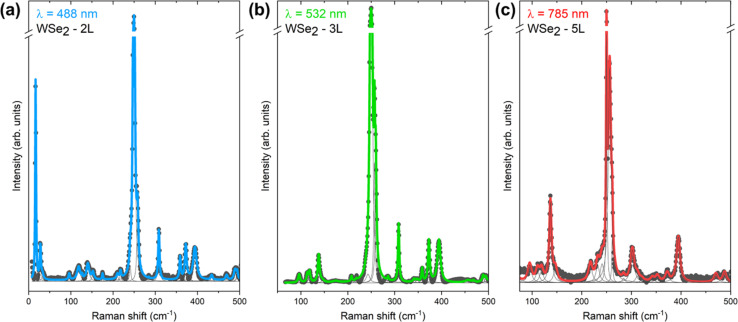
Lorentzian deconvolution of the Raman spectra for (a) 2L WSe_2_ measured at 488 nm, (b) 3L WSe_2_ measured at 532 nm and (c) 5L WSe_2_ measured at 785 nm excitation wavelengths.

Deconvolution of the Raman spectra measured with 488, 532 and 785 nm excitation for each individual layer system of WSe_2_ was performed using the minimum number of Lorentzian components, allowing the identification of around 35 Raman peaks for each system. The representative deconvolution results corresponding to Raman spectra of 2L WSe_2_ measured at 488 nm, 3L WSe_2_ measured at 532 nm and 5L WSe_2_ measured at 785 nm are shown in Fig. 4a–c. Each peak was modeled with a Lorentzian curve characterized by peak position, peak width, and intensity. As the fitting procedure includes a large number of variables, rigorous restrictions were imposed on the fitting parameters in order to avoid correlation among the parameters and obtain meaningful results, as explained here below and in ref. 36,46–48. This includes leaving the intensity and peak position as free parameters, while the widths of peaks were restricted. As the peak widths are mostly dependent on the phonon lifetime, which is determined by the crystal quality of the material, it is expected that all fundamental one-phonon Raman modes have similar widths, regardless of the symmetry of the mode. This results in allowing only a narrow interval of change for the one-phonon peak widths during the deconvolution process. Possible two-phonon or multi-phonon modes are similarly modeled with higher widths compared to the one-phonon modes. This allows unambiguous interpretation of the phonon nature of the peaks, rendering the identification procedure more accurate.

The Raman frequencies of all peaks obtained from the deconvolution process are listed in Table 1, along with their symmetry assignment and a comparison with the frequency of the DFT calculated phonon modes and experimentally reported values from the literature. All peaks are identified as either one-phonon or multi-phonon modes based on the results from the deconvolution procedure. Overall, we observe excellent agreement (on-average 2% difference) between the experimentally observed peaks and the theoretically predicted Raman frequencies. Minor disagreement in the Raman peak positions between the experimental and the theoretical results is expected, due to approximations applied during the calculations, such as the three-body and long-range interactions.

**Table 1:** Frequency (in cm^−1^) of peaks from Lorentzian deconvolution of the Raman spectra corresponding to 1L–5L and bulk WSe_2_ measured with 488 nm, 532 nm and 785 nm excitation wavelengths^a^

This work							Literature	
*λ*_ext_ (nm)	*ν*_exp_ (cm^−1^)					Symmetry assignment	*ν*_theory_ (cm^−1^)	*ν*_exp_ (cm^−1^)
	1L	2L	3L	5L	Bulk			
**488**	**—**	**28**	**19**	**13**	**—**	**A**_**1g**_ **(even),** 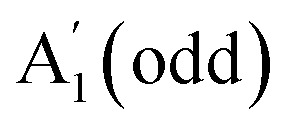 **,** and **B**_**2g**_**(bulk)**	27.7 (2L), 19.4 (3L), and 14.8 (5L)^43^	28 (2L), 19 (3L), and 12 (5L)^43^
**488**	**—**	**17**	**21**	**22**	**24**	**E**_**g**_ **(even), E′ (odd),** and **E**_**2g**_**(bulk)**	17.8 (2L), 21.6 (3L), 23.5 (5L), and 24.6 (bulk)^43^	17 (2L), 21 (3L), 23 (5L), and 24 (bulk)^43^
**488**	**—**	**—**	**—**	**32**	**—**	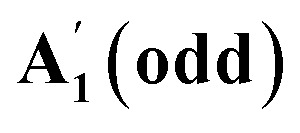	30.7 (5L)^43^	32 (5L)^43^
488	32	36	36	36	35	LA(M) – TA(M)	31.7 (ref. 33)	—
488	75	75	75	75	75	ZO_2_(M)–TO_1_(M)	76.3 (ref. 33)	74.4 (ref. 33)
All	97	97	97	97	97	LO_1_(M)–ZA(M)/TO_1_(M)–TA(M)	94.2/95.3 (ref. 33)	96.0 (ref. 33)
All	115	114	114	111	111	TO_2_(K)–TA(K)/ZO_1_(K)–LA(K)/LO_2_(M)–LA(M)/LO_1_(K)–TA(K)/LO_2_(K)–LA(K)	114.5/114.9/115.1/116.3/116.5 (ref. 33)	114.3 (ref. 33)
All	119	119	119	119	119	ZO_2_(K)–LA(K)	119.5 (ref. 33)	120.1 (ref. 33)
All	128	128	128	127	127	TO_2_(M)–TA(M)	130.7 (ref. 33)	130.5 (ref. 33)
All	138	138	138	138	138	LO_2_(K)–ZA(K)/ZO_1_(*Γ*)–TO/LO_1_(*Γ*)	135.2/136.2 (ref. 33)	137.4 (ref. 33)
488, 532	147	147	147	147	147	LO_2_(M)–TA(M)	146.8 (ref. 33)	145.0 (ref. 33)
All	153	153	153	153	153	ZO_1_(K)–TA(K)	157.4 (ref. 33)	158.4 (ref. 33)
**488, 532**	**—**	**176**	**175**	**175**	**176**	**E**_**g**_ **(even), E′ (odd),** and **E**_**1g**_**(bulk)**	176.3 (2L)^27^	176.3 (2L)^27^
488, 532	209	209	209	209	209	3- Or 4-phonon mode (example: TO_2_(M)–TA(M) + ZO_2_(M)–TO_1_(M))	209.6 (ref. 33)	208.1 (ref. 33)
All	219	219	219	218	219	TA(M) + ZA(M)	220.4 (ref. 33)	219.2 (ref. 33)
532, 785	229	229	229	232	232	TA(M) + LA(M)	228.5 (ref. 33)	231.2 (ref. 33)
All	240	240	240	241	241	TA(K) + LA(K)	242.3 (ref. 33)	239.9 (ref. 33)
**All**	**250**	**250**	**250**	**250**	**250**	**E**_**g**_ **& A**_**1g**_ **(even), E′ &** 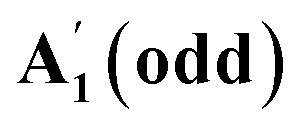 **,** and **E**_**2g**_**& A**_**1g**_**(bulk)**	250.8 & 251.3 (ref. 33)	249.9 (ref. 33)
All	258	258	258	257	257	2vHs	256 (ref. 33)	258.7 (ref. 34)
All	261	261	261	261	261	2LA(M)	260.2 (ref. 33)	261.0 (ref. 33)
488, 532	283	283	284	284	284	2LA(K)	284.8 (ref. 33)	274.1 (ref. 34)
**All**	**—**	**309**	**309**	**309**	**309**	**A**_**1g**_ **(even),** 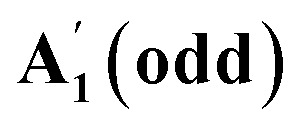 **,** and **B**_**2g**_**(bulk)**	311.3 (ref. 27)	310 (ref. 27)
488, 532	331	330	330	331	331	TO_1_(K) + ZA(K)	333.3 (ref. 33)	331.3 (ref. 33)
488, 532	340	341	341	341	341	TO_2_(M) + ZA(M)	339.9 (ref. 33)	341.5 (ref. 33)
All	351	350	350	352	352	2 TO/LO_1_(*Γ*)	352.3 (ref. 33)	351.2 (ref. 33)
488, 532	359	359	359	359	359	ZO_1_(M) + TA(M)/ZO_2_(K) + TA(K)	361.7/361.8 (ref. 33)	360.0 (ref. 33)
All	374	373	373	373	373	LO_2_(M) + LA(M)	373.3 (ref. 33)	375.4 (ref. 33)
All	396	394	394	394	394	ZO_2_(M) + ZA(M)	392.0 (ref. 33)	393.0 (ref. 33)
532	444	444	444	—	—	LO_1_(M) + TO_2_(M)	445.3 (ref. 33)	—
488, 532	456	456	456	456	456	TO_1_(M) + ZO_1_(M)/2TO_2_(M)/LO_2_(M) + TO_2_(M)	457.0/458.2/461.4 (ref. 33)	—
488, 532	469	469	469	469	469	TO_1_(K) + ZO_2_(K)/TO_2_(K) + ZO_1_(K)	471.5/471.7 (ref. 33)	—
All	488	488	488	488	488	LO_1_(*Γ*) + ZO_1_(*Γ*)/ZO_1_(M) + TO_2_(M)	488.29/492.4 (ref. 33)	491.3 (ref. 33)
488, 532	496	496	496	496	496	ZO_2_(M) + TO_2_(M)	499.1 (ref. 33)	499.2 (ref. 33)
488, 532	510	511	511	512	512	ZO_1_(M) + LO_2_(M)/2ZO_1_(K)	508.5/514.6 (ref. 33)	507.1/514.4 (ref. 33)
All	533	532	532	532	532	ZO_1_(M) + ZO_2_(M)	533.3 (ref. 33)	533.8 (ref. 33)

a Labels: *λ*_ext_ – laser excitation under which the Raman mode is present; *ν*_exp_ – Raman peak frequency experimentally determined from the Raman spectra; *ν*_theory_ – theoretically calculated Raman mode frequency; “ – ” Raman peak not observed in the spectra; “/” – additional phonon mode assignment for the Raman peak with the same frequency. Fundamental one-phonon Raman modes arising from the *Γ* point are highlighted in bold.

### First-order Raman modes in multilayer and bulk WSe_2_

First, we focus on the identification of the fundamental, one-phonon modes arising from the *Γ* point as the result of first-order Raman scattering. For convenience, in the text, we use the symmetry notation of bulk WSe_2_, while the corresponding symmetry mode labeling for different numbers of layers is presented in Table 1. One-phonon Raman peaks are located at around 10–35, ∼176, ∼250, and ∼309 cm^−1^. The peak at 250 cm^−1^ is the most intense peak in WSe_2_ systems, present for all numbers of layers and corresponds to the overlapped E_2g_ and A_1g_ phonons. Although the energies of these phonons are not degenerate (see theoretical values in Table 1), the two peaks cannot be distinguished as their energy difference is below the resolution limit of the setup. The peaks at 176 and 309 cm^−1^ are identified as E_1g_ and B_2g_ modes, respectively, and are present for all thicknesses, except for the monolayer. No change in the frequency of all the above mentioned peaks for different numbers of layers is observed within the resolution of our Raman measurements (<2 cm^−1^). This is in agreement with the DFT and previous experimental reports.^25,27–29,33,34,43–45^ Next, several low Raman intensity peaks can be observed in the region from 10 to 35 cm^−1^. These peaks are identified as interlayer breathing and shear modes with B_2g_ or E_2g_ symmetries and frequencies depending on the number of layers.^43^ These modes are a consequence of the interlayer van der Waals (vdW) coupling between the layers and are therefore not present in the case of monolayer WSe_2_.

### Second/higher-order Raman modes in multilayer and bulk WSe_2_

The other peaks present in the Raman spectra of WSe_2_ systems are identified as two-phonon modes from second-order Raman scattering. These modes arise from the combination of two phonons at the *Γ*, *M* or *K* points of the Brillouin zone, whose total momentum is zero. The combination modes have been identified based on the frequencies of the individual phonons, calculated from the DFT obtained phonon dispersions for 1L to 3L WSe_2_, presented in ref. 33. Overall, there is a very good agreement between the theoretically predicted and experimentally observed frequencies of modes in all cases (Table 1), except for the peak at 209 cm^−1^. This peak was previously identified as a single phonon mode arising from the *K* point of the TO_1_ phonon branch.^33^ However, activation of one-phonon modes away from the *Γ* point in the Brillouin zone (such as *M* and *K* points) requires the presence of a defect, which will nullify the momentum of the phonon, therefore preserving the total momentum conservation law. As this mode appears in the Raman spectra of all layer systems, including the bulk, it would mean that there is a systematic structural defect present in the lattice of WSe_2_ regardless of the number of layers or position on the flake. While this could not be completely excluded, a more probable process, such as multiphoton scattering should also be considered. Based on the low intensity of this peak, we believe that it might be arising from a multi Raman scattering process involving three or more phonons, such as TO_2_(M) − TA(M) + ZO_2_(M) − TO_1_(M). We also assign the peak at 258 cm^−1^ to the scattering of two phonons due to a van Hove singularity (vHs)^34,49,50^ at the saddle-point in the phonon density of states, located between the *K* and *M* points of the LA phonon branch (Fig. S2 in the ESI†) and labeled as 2vHs in Table 1.

Finally, we note the appearance of three peaks at 302, 432 and 520 cm^−1^ in the Raman spectra of all WSe_2_ systems. These peaks arise from the oxidized Si substrate, as shown in the Raman spectra of the bare substrate in Fig. S3(a) in the ESI.† The peak at 302 cm^−1^ should be especially mentioned, as it has been previously attributed to WSe_2_,^30^ and explained as experiencing Breit–Wigner–Fano (BWF) type resonance for Raman measurements with 785 nm excitation. We disagree with this explanation as (1) there is no theoretically predicted one-phonon or multi-phonon mode of WSe_2_ at this frequency, (2) the Raman intensity of this peak scales exactly the same as the Raman intensity of the bulk Si 520 cm^−1^ peak (Fig. S3(b) in the ESI†), and (3) no other Raman modes of WSe_2_, especially fundamental one-phonon modes, experience BWF resonance for measurements with 785 nm, which makes it highly unlikely that only the 302 cm^−1^ mode would have such behavior. In conclusion, we attribute the Raman peak at 302 cm^−1^ to the TA phonon of Si and not to WSe_2_.

### Intensity amplification in Raman scattering of multilayer and bulk WSe_2_

**Fig. 5:**
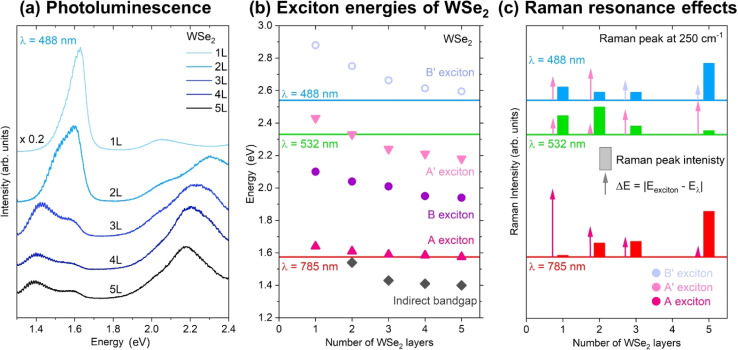
Exciton energies of WSe_2_ and Raman resonance. (a) Room-temperature photoluminescence spectra of 1L–5L of WSe_2_ measured at 488 nm excitation wavelength. (b) Exciton energies in 1L–5L WSe_2_ determined from the PL peak positions corresponding to the indirect band gap, A, B, A′ and B′ excitons. It should be noted that the energy values of the B′ exciton have been taken from ref. 51. Energies of the laser excitations used for the Raman measurements are indicated with horizontal lines labeled 488, 532 and 785 nm. (c) Comparison of the Raman intensity of the 250 cm^−1^ peak with the energy difference between WSe_2_ excitons and laser excitation, allowing identification of Raman resonance conditions for certain WSe_2_ flake thicknesses.

The variation of the excitation wavelength in Raman scattering provides a path to correlate the phonon with the energy band structure of the material. The identification of wavelengths maximizing the scattered intensity, also known as resonant, enables measurements with minimum excitation intensity. While our study does not include a fully tunable laser, we use multiple wavelengths. Some conditions result in a significant increase in the scattered intensity, which we relate to the band structure of our material. In order to understand the potential appearance of amplification conditions when using different excitation wavelengths, room-temperature photoluminescence (PL) measurements have been performed on the multilayer WSe_2_ using a 488 nm excitation wavelength, as shown in Fig. 5a. Overall, a total of four PL contributions are observed in the PL spectra of 1–5L WSe_2_. These have been attributed to the indirect bandgap at around 1.4 eV (2L–5L systems only), the A exciton at around 1.65 (1L), the B exciton at around 2.1 eV (1L) and the A′ exciton at 2.4 eV (1L). A, B and A′ excitons shift to lower energies with the increase in the flake thickness from 1L to 5L, as shown in Fig. 5b. Fig. 5b also plots the position of the B′ exciton obtained from the absorption measurements reported in ref. 51, which is not accessible to our PL measurements. A detailed explanation of the origin of these excitonic states was reported in several studies^25,52–57^ and it is not the focus of this work.

The comparison of the Raman scattering intensity of the A_1g_/E_2g_ mode located at 250 cm^−1^ with the energy difference between a certain exciton and laser excitation is plotted in Fig. 5c. In the case of the 488 nm laser excitation, we can observe that the Raman intensity of the A_1g_/E_2g_ mode is high for 1L, decreases for 2L and 3L and again increases for 5L WSe_2_. Considering the band structure of WSe_2_, amplification effects due to allowed electronic transitions are observed in the case of 1L. They are due to the coupling with the A′ exciton, and in the case of 5L, due to coupling with the B′ exciton, as shown by the line indicating the laser energy in Fig. 5b. For Raman measurements performed with 532 nm excitation, the highest intensity of the A_1g_/E_2g_ mode is observed in the case of 2L WSe_2_. This is followed by slightly lower intensities for 1L and 3L and the lowest intensity for 5L WSe_2_. The Raman amplification in this case is due to coupling with the A′ exciton, whose energy in the case of 2L WSe_2_ (2.32 eV) ideally coincides with the green laser excitation (2.34 eV). Finally, the Raman amplification in the case of 785 nm laser excitation is observed for 5L WSe_2_, as the intensity of the A_1g_/E_2g_ mode is the highest in comparison to the other thicknesses. The Raman amplification in this case is due to coupling with the A exciton, whose energy for 5L WSe_2_ is around 1.59 eV, which is very close to the energy of the red laser excitation (1.58 eV). This implies that further Raman characterization, where, for example phonon–phonon or electron–phonon interactions would be investigated, would be best achieved when using 488 nm excitation for 1L and 3L, 532 nm excitation in the case of 2L and 785 nm excitation for 5L and higher WSe_2_ thicknesses.

### Phonon cascades in multilayer and bulk WSe_2_

We now turn to the low wavenumber domain of the Raman spectra, which contains information on phonon cascade processes. Phonon cascade processes occur in the first stages of carrier recombination. They are particularly fast in TMDs, rendering pump-probe experiments challenging.^58^ In this sense, Raman spectroscopy close to the Rayleigh line provides relevant information on these processes. A fundamental understanding and characterization of these processes is key for their application in optoelectronics.

**Fig. 6:**
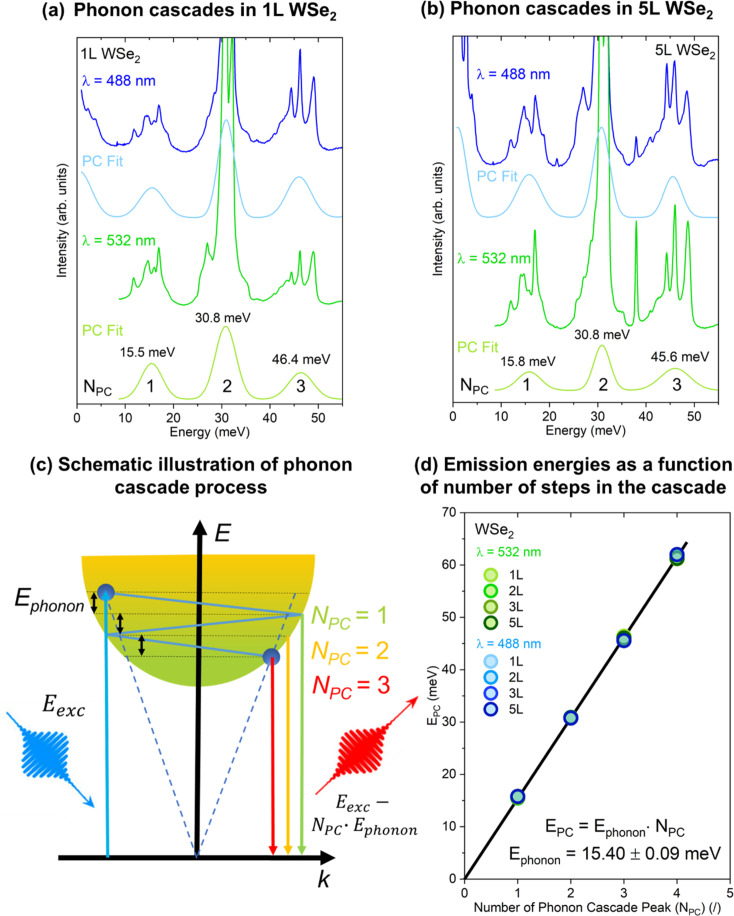
Phonon cascades in WSe_2_: Raman spectra of (a) 1L and (b) 5L WSe_2_ measured with 488 and 532 nm laser excitations, with phonon cascade fit (labeled as PC Fit) revealing 3 periodic Gaussian peaks, labeled as *N*_PC_ = 1, 2 and 3, at about 16, 31 and 46 meV, respectively. (c) Schematic illustration of the phonon cascade process. (d) Emission energies of PC peaks as a function of the number of steps in the cascade obtained from the PC fitting of the Raman spectra of 1L–5L WSe_2_ measured with laser excitations of 488 and 532 nm.

Fig. 6a and b present the Raman spectra of 1L and 5L WSe_2_ respectively, measured with 488 and 532 nm excitations, along with the phonon cascade fits. We attribute these peaks to phonon cascades (PCs) in the Raman spectra of 1L–5L and bulk WSe_2_. These oscillations are not present under 795 nm excitation, as can be clearly seen in Fig. 3f, upon comparison with Fig. 3c and d. We have fit the spectra with three periodic Gaussian peaks, labeled as *N*_PC_ = 1, 2 and 3, at about 16, 31 and 46 meV, respectively. Fittings of Raman spectra for 2L and 3L layers are presented in Fig. S4 in the ESI.†

Phonon cascades^59,60^ are due to the electron–phonon interaction, where electrons undergo phonon-induced transitions with a radiative recombination at each step, as illustrated in Fig. 6c. Phonon cascades start when incoming photons with energy (*E*_exc_) excite electrons from the valence band to energy states corresponding to the in-plane wavevector cone of *k* ≤ *ω*_exc_/*c*, where *ω*_exc_ is the frequency of the incoming photons (this cone is indicated with blue dashed lines in Fig. 6c). Due to phonon–electron interaction, the excited electrons undergo successive transitions between vibrational states (labeled with dashed black lines in Fig. 6c). At each step, one phonon with an energy corresponding to the difference between the vibrational states (*E*_phonon_) is created. The creation of phonons is followed by a possible radiative recombination of the electron with the hole in the valence band and emission of a photon with an energy *E*_exc_ – *N*_PC_*E*_phonon_, where *N*_PC_ corresponds to the number of phonons that were created in the phonon cascade process. As a result, periodic emission peaks are observed in the energy region close to the laser excitation (*i.e.* the Raman region, also called the hot photoluminescence region). According to the theory,^61^ similar behavior of phonon cascades is expected for involvement of excitons, as well as for free electrons or holes. Therefore, it is not possible to distinguish between free-carrier and exciton cascades from hot photoluminescence experiments. However, considering that excitonic behavior is dominant in WSe_2_, it makes sense that phonon cascades involving excitons are more probable.

The excitation energy of the incoming photons needs to be above the free-carrier gap excitation for phonon cascades to occur. This is the reason why phonon cascades in WSe_2_ are not observed with 785 nm laser excitation, as the energy of the A exciton for 1L–5L WSe_2_ is always higher than the 785 nm laser energy (1.58 eV), which in turn is insufficient to excite the carriers above the A exciton level.

Previously, phonon cascades were reported only in monolayer WSe_2_.^62^ Here, we report phonon cascades up to 5L and bulk WSe_2_. While the fine structure of the spectra is slightly different as a function of the number of ML, PC fitting did not reveal any significant shift in the energy of the PC peaks with the change in the WSe_2_ layer thickness, or with the change in the laser excitation, as indicated in Fig. 5d. Linear fitting of the PC peak energy (*E*_PC_) with the number of peaks in the cascade (*N*_PC_), allows for the determination of the energy of the phonon (*E*_phonon_) involved in the cascade process, as shown in Fig. 5d. This also corresponds to the average energy difference between successive PC peaks. In this case, the obtained phonon energy of around 15.4 meV is in agreement with the reports for monolayer WSe_2_.^62^ This energy corresponds well to the out-of-plane (ZA) acoustic phonon (around 125 cm^−1^) at the *M* and *K* points of the Brillouin zone (Fig. S2 in the ESI†). The involvement of the ZA phonon is further confirmed by its phonon branch being relatively flat in the region between the *M* and *K* points, which is necessary for the generation of a high number of PC peaks.

The observation of phonon cascades in WSe_2_ indicates that an exceptionally strong electron–phonon interaction occurs in the initial steps of carrier relaxation that prevails until 5ML, which may provide design-relevant insights for emerging optoelectronic applications.

## Conclusions

We present a complete investigation of the vibrational properties of 1L to 5L and bulk WSe_2_ using multiwavelength excitation Raman spectroscopy. The systematic deconvolution and comprehensive analysis of Raman spectra across different excitation energies enabled the reliable identification of 35 Raman peaks for each flake thickness, which were attributed to either one-phonon or two-phonon modes. The results are in agreement with the theoretical predictions. Furthermore, we have resolved inconsistencies in the literature in terms of activation of non-*Γ* point single phonon modes and BWF type resonance. Using photoluminescence measurements, we identify photon-exciton coupling leading to strong enhancement in Raman intensity. Based on this, we suggest optimal laser excitations best suited for further Raman investigations of specific WSe_2_ layer thicknesses. Finally, we report the observation of phonon-cascades involving ZA phonons for all WSe_2_ layer thicknesses, indicating strong phonon–electron interactions during early carrier relaxation processes. This research provides a solid foundation and reference for further investigations of the vibrational properties of WSe_2_, paving the way for WSe_2_ integration into advanced optoelectronic and phononic devices.

## Data availabitlity

The data for this article are available at https://doi.org/10.5281/zenodo.12696856.

## Author contributions

M. D. and A. F. iM. conceived the research idea and supervised the work. C. B. performed the Raman and AFM measurements. A. B. prepared the sample under supervision of M. B. M. D. performed the PL measurements. C. B., A. L. and M. D. analyzed the data. M. D. and C. B. wrote the manuscript with inputs from all co-authors.

## Conflicts of interest

The authors declare that they have no competing financial interests.

## Supplementary Material

NA-OLF-D4NA00399C-s001
