# Supplementary material for: Unveiling the complex phonon nature and phonon cascades in 1L to 5L WSe2 using multiwavelength excitation Raman scattering
Source: Nanoscale Adv. 2024 Jul 30;6(18):4591–603. doi: 10.1039/d4na00399c (PMC11325308; doi:10.1039/d4na00399c)
Supplement: NA-006-D4NA00399C-s001 [file NA-006-D4NA00399C-s001.pdf]

Supplementary Information (SI) for Nanoscale Advances.
This journal is © The Royal Society of Chemistry 2024

**Supporting Information for:**

# Unveiling the Complex Phonon Nature and Phonon Cascades in 1L to 5L $WSe_2$ Using Multiwavelength Excitation Raman Scattering

Claire Blaga,[1] Angel Labordet Alvarez,[2] Akshay Balgarkashi,[1] Mitali Banerjee,[3] Anna Fontcuberta i Morral,[1,3] Mirjana Dimitrievska[1,2*]

*1 - Laboratory of Semiconductor Materials, Institute of Materials, School of Engineering, Ecole Polytechnique Fédérale de Lausanne, 1015, Lausanne, Switzerland*

*2 - Transport at Nanoscale Interfaces Laboratory, Swiss Federal Laboratories for Material Science and Technology (EMPA) Ueberlandstrasse 129, 8600 Duebendorf, Switzerland*

*3 - Laboratory of Quantum Physics, Topology and Correlations, Institute of Materials, School of Engineering, Ecole Polytechnique Fédérale de Lausanne, 1015, Lausanne, Switzerland*

*4 - Institute of Physics, School of Basic Sciences, Ecole Polytechnique Fédérale de Lausanne, 1015, Lausanne, Switzerland*

**corresponding author:* mirjana.dimitrievska@empa.ch

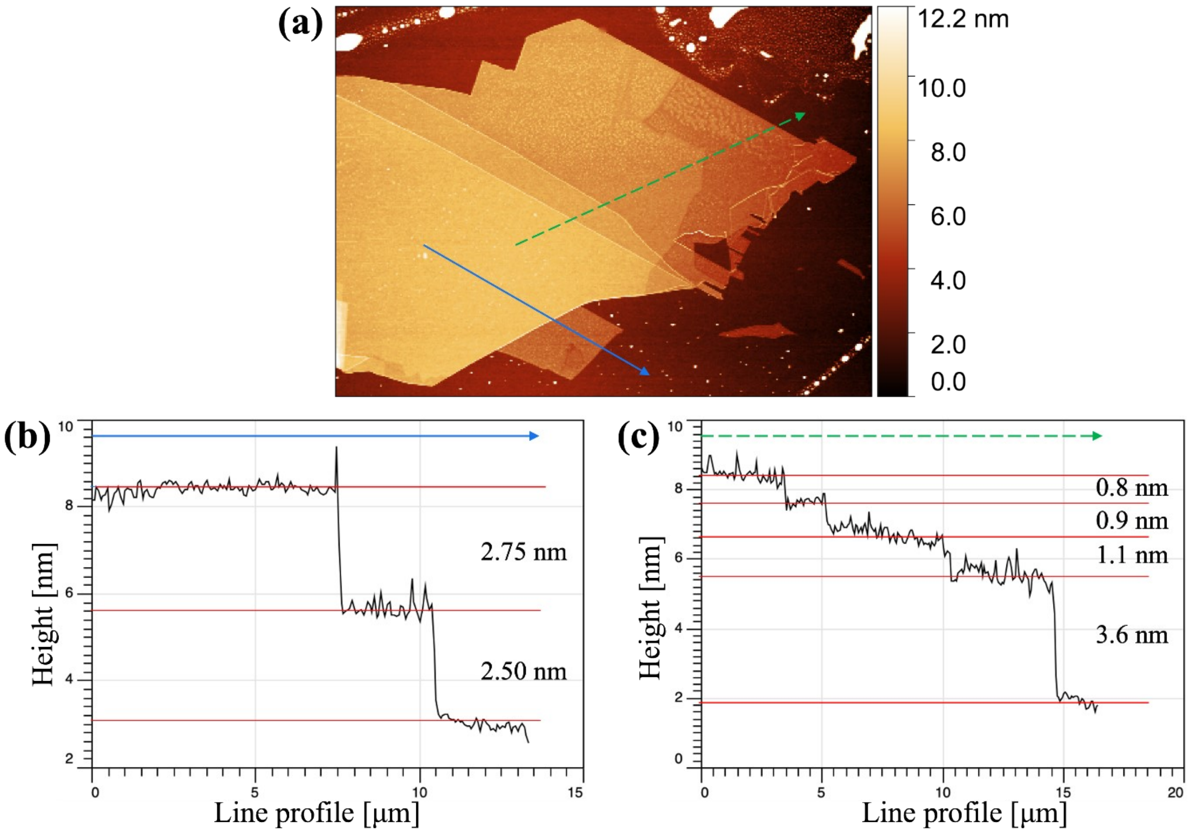


**Figure S1.** AFM characterization of $WSe_2$ flake. (a) AFM image of the $WSe_2$ flake measured in tapping-mode showing indicating to line profiles which were used for the identification of the number of layers. (b) and (c) AFM line profiles showing differences in the layer thicknesses and allowing identification of 1L to 5L layers.

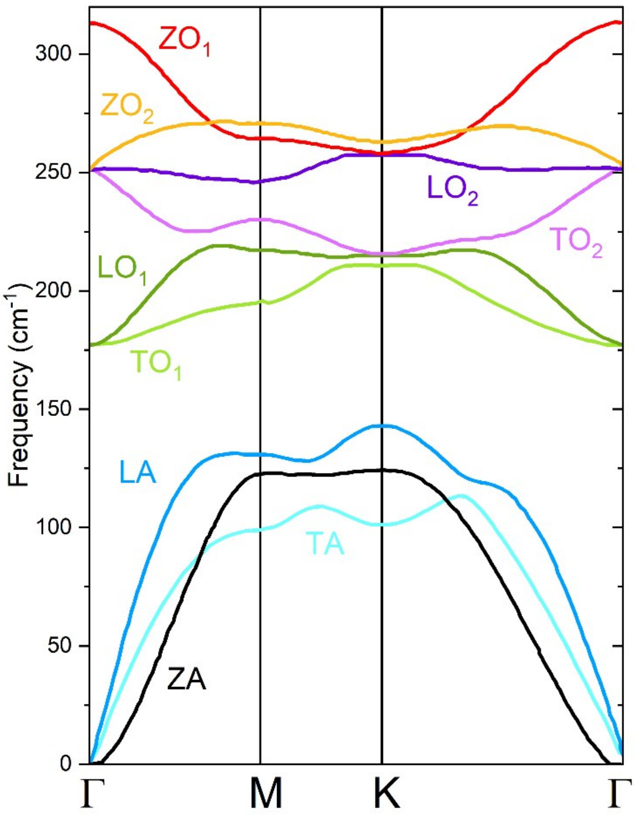


**Figure S2.** Density Functional Theory (DFT) calculated phonon dispersion of monolayer WSe2 adapted from Ref. [1], with labeled phonon branches. Monolayer $WSe_2$ has three atoms per unit cell, resulting in total of nine phonons. The three acoustic phonons correspond to the out of plane (ZA) vibrations, in-plane transverse (TA) vibrations, and in-plane longitudinal (LA) vibrations. These phonons are located at 0 $cm^{-1}$ at the Γ point and correspond to the lowest frequency phonon branches across the Brillouin zone. The E'' and E' phonon modes are located at 176 and 250 $cm^{-1}$ at the Γ point, and as doubly degenerated modes, are split into two optical branches each, over the Brillouin zone. The lower frequency phonon branches correspond to transverse ($TO_1$ and $TO_2$) vibrations, while the higher frequency phonon branches correspond to longitudinal ($LO_1$ and $LO_2$) vibrations of E'' and E' modes, respectively. The $A_1$' and $A_2$'' modes are located at 250 and 310 $cm^{-1}$ at the Γ point and correspond to two out of plane ($ZO_1$ and $ZO_2$) vibrations, respectively. They are represented with the highest frequency phonon branches.

[1] M. D. Luca, X. Cartoixà, J. Martín-Sánchez, M. López-Suárez, R. Trotta, R. Rurali and I. Zardo, New insights in the lattice dynamics of monolayers, bilayers, and trilayers of WSe2 and unambiguous determination of few-layer-flakes' thickness, *2D Mater.*, 2020, **7**, 025004.

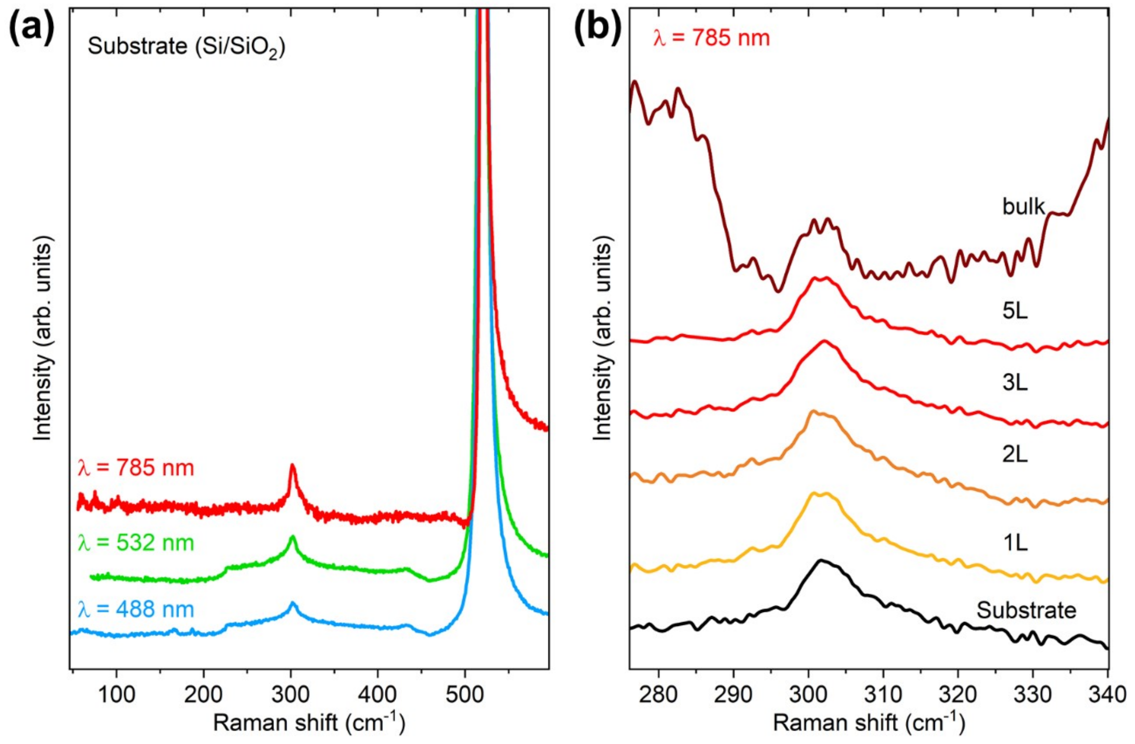


**Figure S3. (a)** Raman spectra of bare $Si/SiO_2$ substrate measured with 488, 532 and 785 nm laser excitations allowing easier discrimination of Raman peaks attributed to the substrate. (b) Comparison of 302 $cm^{-1}$ Raman peak of 1L to 5L and bulk $WSe_2$, to bare $Si/SiO_2$ substrate. All Raman spectra were measured with 785 nm laser excitation and normalized to Si (520 $cm^{-1}$) peak.

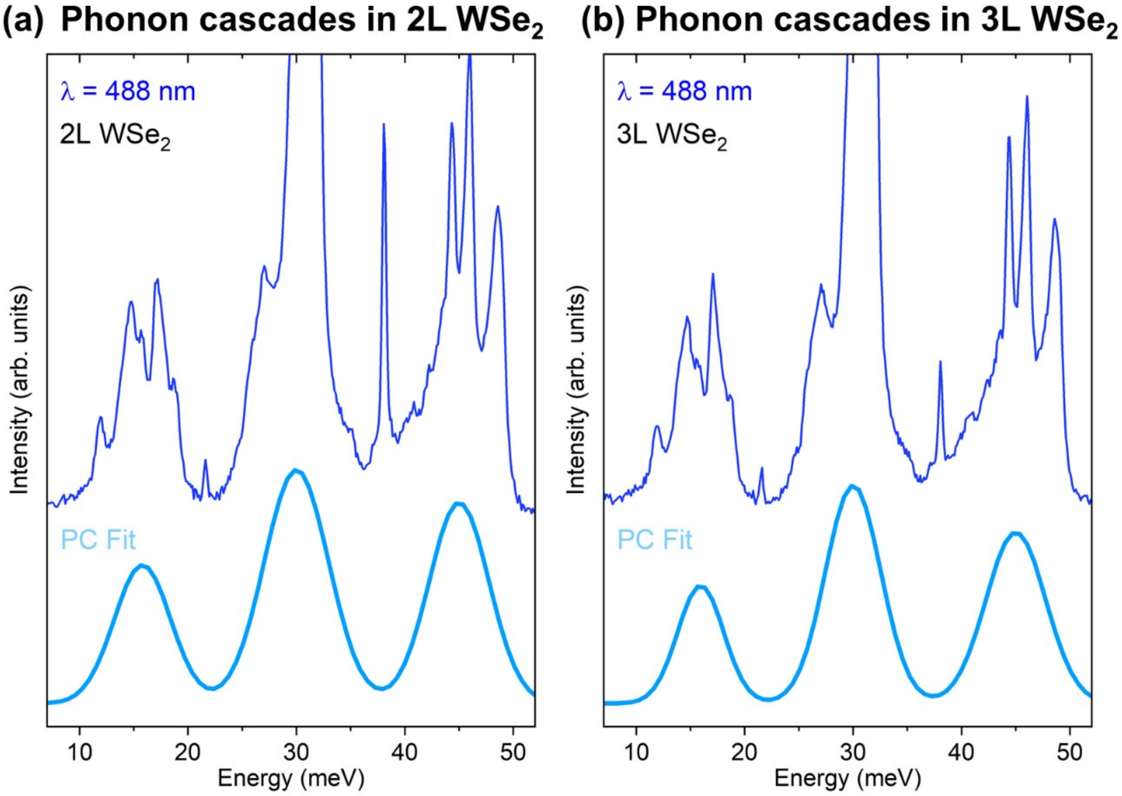


**Figure S4.** Raman spectra of **(a)** 2L and **(b)** 3L $WSe_2$ measured with 488 laser excitation, with phonon cascade fit (labeled as PC Fit) revealing 3 periodic Gaussian peaks, labeled as $N_{PC}$ = 1, 2 and 3, at about 16, 31 and 46 meV, respectively.
